# Exposure to a multi-level multi-component childhood obesity prevention community-randomized controlled trial: patterns, determinants, and implications

**DOI:** 10.1186/s13063-018-2663-y

**Published:** 2018-05-22

**Authors:** Angela Cristina Bizzotto Trude, Anna Yevgenyevna Kharmats, Jessica C. Jones-Smith, Joel Gittelsohn

**Affiliations:** 1 Global Obesity Prevention Center and Center for Human Nutrition, Department of International Health, Johns Hopkins Bloomberg School of Public Health, 615 N. Wolfe Street, Baltimore, MD 21205 USA; 2 Nutritional Sciences Program, University of Washington School of Public Health University of Washington, 353410, 306B Raitt Hall, Seattle, WA USA

**Keywords:** Process evaluation, Childhood obesity, Dose received, Methods, Implementation process, Exposure

## Abstract

**Background:**

For community interventions to be effective in real-world conditions, participants need to have sufficient exposure to the intervention. It is unclear how the dose and intensity of the intervention differ among study participants in low-income areas. We aimed to understand patterns of exposure to different components of a multi-level multi-component obesity prevention program to inform our future impact analyses.

**Methods:**

B’more Healthy Communities for Kids (BHCK) was a community-randomized controlled trial implemented in 28 low-income zones in Baltimore in two rounds (waves). Exposure to three different intervention components (corner store/carryout restaurants, social media/text messaging, and youth-led nutrition education) was assessed via post-intervention interviews with 385 low-income urban youths and their caregivers. Exposure scores were generated based on self-reported viewing of BHCK materials (posters, handouts, educational displays, and social media posts) and participating in activities, including taste tests during the intervention. For each intervention component, points were assigned for exposure to study materials and activities, then scaled (0–1 range), yielding an overall BHCK exposure score [youths: mean 1.1 (range 0–7.6 points); caregivers: 1.1 (0–6.7), possible highest score: 13]. Ordered logit regression analyses were used to investigate correlates of youths’ and caregivers’ exposure level (quartile of exposure).

**Results:**

Mean intervention exposure scores were significantly higher for intervention than comparison youths (mean 1.6 vs 0.5, *p* < 0.001) and caregivers (mean 1.6 vs 0.6, *p* < 0.001). However, exposure scores were low in both groups and 10% of the comparison group was moderately exposed to the intervention. For each 1-year increase in age, there was a 33% lower odds of being highly exposed to the intervention (odds ratio 0.77, 95% confidence interval 0.69; 0.88) in the unadjusted and adjusted model controlling for youths’ sex and household income.

**Conclusion:**

Treatment effects may be attenuated in community-based trials, as participants may be differentially exposed to intervention components and the comparison group may also be exposed. Exposure should be measured to provide context to impact evaluations in multi-level trials. Future analyses linking exposure scores to the outcome should control for potential confounders in the treatment-on-the-treated approach, while recognizing that confounding and selection bias may exist affecting causal inference.

**Trial Registration:**

ClinicalTrials.gov, NCT02181010. Retrospectively registered on 2 July 2014.

**Electronic supplementary material:**

The online version of this article (10.1186/s13063-018-2663-y) contains supplementary material, which is available to authorized users.

## Background

The multifactorial causes of obesity are well recognized, and it is clear that no single community program or policy provides a comprehensive solution to this important public health problem [1–3]. Thus, it is suggested that multi-level multi-component (MLMC) interventions are more effective than single-component interventions, due to synergistic effects between multiple intervention components [4, 5]. However, to achieve these effects, MLMC interventions need to reach the population of interest in sufficient intensity, i.e., achieve adequate exposure. Therefore, the evaluation of a program’s implementation needs to be systematically measured and evaluated, as it informs research into practice gaps [6], allowing replication in real-world settings and large-scale public health dissemination [7].

Given the importance of implementation to study outcomes, a growing body of literature has used process evaluation to assess intervention fidelity (adherence to intervention protocol) [8–10]. Process evaluation is used to monitor and improve program delivery and helps explain reasons for failure or success of a trial [11]. Among process evaluation constructs, exposure (dose received) is rarely measured, but it allows researchers to understand how well a program has reached its intended audience from the participants’ perception of their personal exposure and the extent to which they actively engaged with the research activities and materials [12].

Few studies have reported assessing the extent to which individual study participants report being exposed to the intervention. A previous environmental obesity intervention trial, Navajo Health Stores in American Indian reservations, developed exposure scores based on participants’ report of study dose received and found a negative correlation with body mass index (BMI) changes [13]. Another multisite childhood obesity trial, Child and Adolescent Trial for Cardiovascular Health (CATCH), used exposure questionnaires to improve the specificity of the intervention and elicit more specific information on intervention components [14]. Baltimore Healthy Eating Zones (BHEZ), a childhood obesity prevention trial conducted at multiple levels in the low-income urban area of Baltimore City, used different components of the intervention to create an exposure score to inform a secondary evaluation analysis [15]. The Boost program, a Danish multi-component school-based trial, used measures of exposure as the extent of parental involvement in the intervention, and found that students with a high exposure level consumed more fruits and vegetables daily than those with a low exposure score [16].

Despite the different use of exposure measurements, to our knowledge no study has investigated how exposure level varies by participant characteristics, nor has explored differences in exposure levels between both adult caregivers and their child participants in a multi-level intervention. This could be used to inform the study’s external validity and to identify observed confounding characteristics of individuals who may be reached at different doses by the intervention [17].

This study aimed to identify the patterns and determinants of the different levels of exposure to the B’more Healthy Community for Kids (BHCK) intervention, a community-based randomized childhood obesity prevention trial intervening at multiple levels (corner store, carryout, social media/text messaging, and recreation center youth-led nutrition education) of the food system in low-income urban areas of Baltimore City in two rounds (waves) [18]. Specifically, this paper answers the following questions:

1. What were the patterns of exposure (dose received) to BHCK intervention materials and activities among youths and caregivers?
2. How did patterns of overall exposure differ by treatment group among youths and their caregivers?
3. What individual and household factors are associated with exposure to the BHCK intervention?

## Methods

### Design and setting

The BHCK trial was implemented at multiple levels of the urban food environment to improve access to, purchase of, and consumption of healthy food among low-income youths and their families living in food deserts in Baltimore. The BHCK study used a pre- and post-intervention assessment design, with two groups, intervention and comparison, in a 1:1 ratio. The intervention was implemented in two waves (wave 1: August 2014–February 2015 and wave 2: November 2015–August 2016).

The intervention took place in 28 zones, randomized to intervention (*n* = 7 per wave) and comparison groups (*n* = 7 per wave). Assignment occurred publicly by drawing names of eligible recreation centers from a hat. Recreation centers were the nucleus of each zone. A zone’s eligibility criteria in the BHCK trial were: (1) predominantly African American (>50%), (2) low-income neighborhood (>20% of residents living below poverty line), (3) minimum of five small food sources (<3 aisles and no seating), and (4) a recreation center more than 1/2 mile away from a supermarket. A sample of adult caregiver and child dyads were actively recruited at each recreation center and nearby corner stores in the 1.5-mile zone buffer by a BHCK data collector. Household eligibility criteria included: (1) having at least one child aged 10–14 years, (2) having lived in the same location for at least one month, and (3) does not anticipate moving in the next 2 years [18].

A detailed description of the intervention (including formative research, sample size calculation, and intervention activities) is provided elsewhere [18–20]. The BHCK intervention included three main intervention components to target youths and their caregivers:

1. nutrition education and cooking classes (14 lessons of 1-hour duration) led by local youth leaders (intensively trained Baltimore college and high school students), which took place every other week at each intervention community recreation center during after-school programs [19]
2. environmental intervention at the point of purchase in small food stores (corner stores) and family-owned prepared-food outlets (carryout restaurants), which sought to increase stocking of healthier options by store owners and encourage demand of healthful foods through promotions and taste tests of healthier foods and beverages by intervention staff, implemented every other week at intervention corner stores and carryout restaurants for sessions lasting up to 2 hours each [20]
3. social media (Facebook and Instagram) and text-messaging communication campaign, which encouraged goal setting for healthy eating targeted at caregivers, delivered up to three times per week (text messages were sent three times a week and included an interactive component and social media posts were made at least three times per week)

Communications materials (i.e., poster, handouts, educational displays, and giveaways) were professionally developed based on formative research and concurrently displayed during BHCK components and distributed to participants every other week in sequence. The intervention was organized in three themed phases lasting for 2 months each: (1) smart drinks, (2) smart snacks, and (3) smart cooking. All material was aligned with the healthy alternative food product and behavior being promoted at each phase [20].

Study participants were not required to attend recreation center activities nor to visit the BHCK stores and carryouts. However, intervention caregivers were asked if they would like to receive text messages after their baseline appointments and were given a business card with BHCK social media links. In the comparison zones, neither recreation centers nor small food stores received the nutrition education sessions or communication materials, and caregiver–child dyads living in these areas were not enrolled in the BHCK text-messaging program.

### Selection and training of data collectors

Data collectors were trained intensively, through role play and observation training. Group assignments were concealed from the BHCK data collectors who conducted the follow-up assessments. Following the interviews, the data were checked for errors by the interviewer and a second research assistant. The completed questionnaires were examined for missing data or implausible values, and the research team made their best effort to contact the research participants to obtain the needed information.

### Measures

#### Caregiver and youth data collection

Baseline data were collected from July 2013 to June 2014 (wave 1) for a total of 299 youths and their main caregivers (*n* = 298), and from July to December 2015 (wave 2) for 235 caregivers and their youth [18]. The post-evaluation was conducted from March 2015 to March 2016 (wave 1) and from August to January 2017 (wave 2), taking place immediately after implementation of the intervention and running for 1 year (wave 1) or 6 months (wave 2). Youths and caregivers received gift cards after each of the two interviews. Informed assent and consent were gathered from both the youths and caregivers, respectively.

**Fig. 1:**
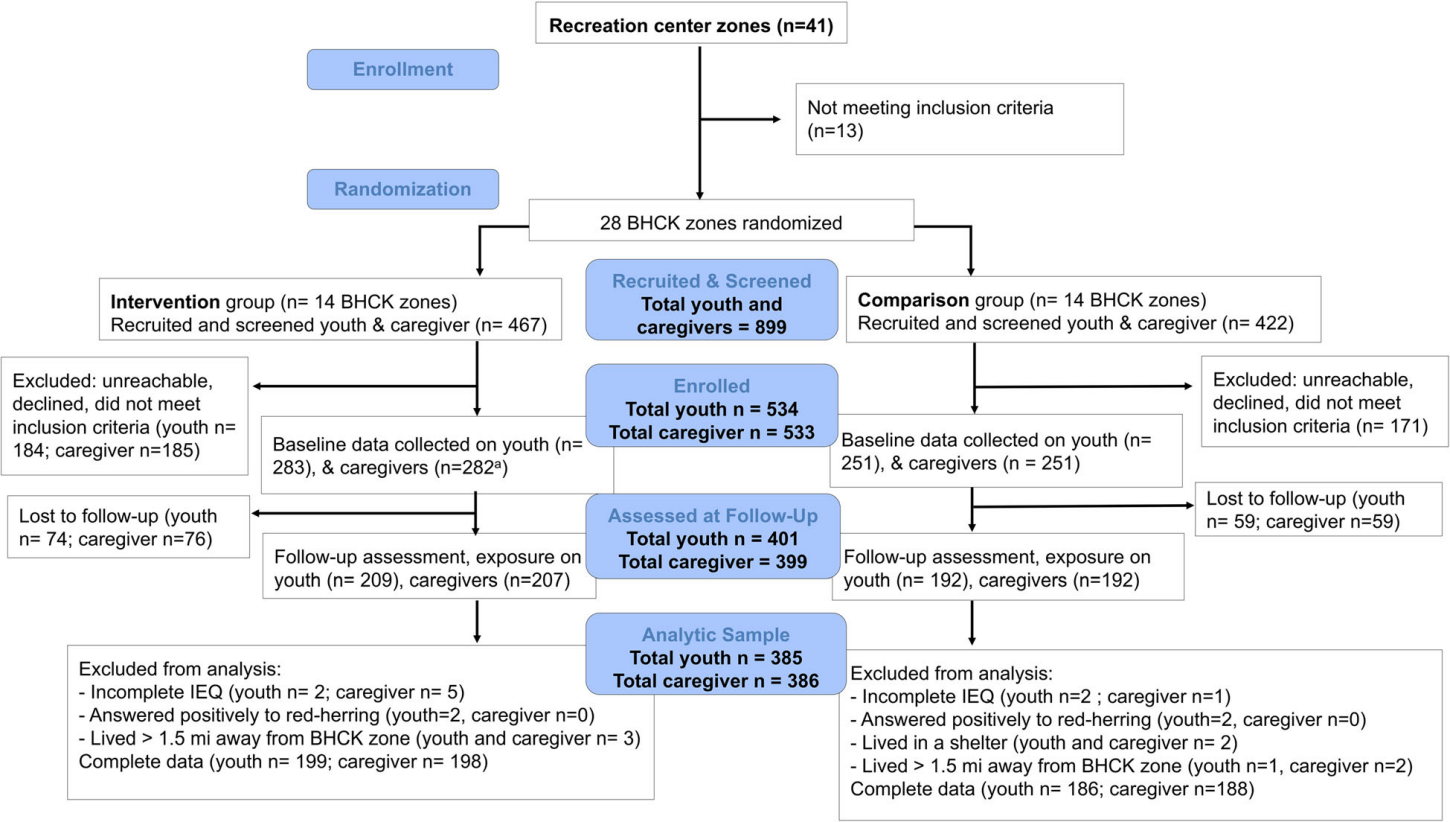
CONSORT flowchart of the randomization and course of the B’more Healthy Communities for Kids program. ^a^One caregiver declined to participate, although they consented to the participation of their youth. *BHCK* B’more Healthy Communities for Kids trial

For the analysis, we excluded participants who did not complete the exposure assessment at the follow-up visit (youths *n* = 133 and caregivers *n* = 135), had missing information for at least one exposure variable (youths *n* = 4 and caregivers *n* = 6), answered positively to more than three of the red-herring questions (youths *n* = 2 and caregivers *n* = 0), reported living in an unstable housing arrangement, such as a shelter or transitional housing (youths *n* = 2 and caregivers *n* = 2), or lived more than 1.5 miles away from a BHCK recreation center (youths *n* = 4 and caregivers *n* = 4). This yielded a total of 385 youths and 386 caregivers for the analytical sample. An overview of enrolment and participant flow is presented in Fig. 1. A CONSORT checklist of information on randomized trials reports is provided as Additional file 1.

#### Process evaluation assessment: exposure (dose received)

The key variables for assessing exposure were obtained from the Intervention Exposure Questionnaire (IEQ) collected as part of the post-intervention assessment for the intervention and comparison groups. To address research questions 1 and 2, we conceptualized the pattern of exposure as the aggregated series of exposures for the reported dose received, to different intervention materials and activities implemented over the course of the program, as assessed by the IEQ. Data collected with the IEQ in wave 1 was used to plan wave 2 intervention modifications [18]. As the waves were implemented 9 months apart, we were able to identify the materials and activities that most of the study sample reported not seeing or not participating in.

The 29-item IEQ included questions to measure exposure to each component of the intervention over the course of program delivery: store and carryout component (16 questions), recreation center (five questions), text messaging (one question), social media (four questions), and program logos and branding (three questions). For visual materials, participants were asked whether they had ever seen the materials during the intervention period (i.e., BHCK logos, posters, handouts, giveaways, educational displays, store shelf labels, and social media posts). For example, the question to assess exposure to BHCK posters was worded: “The BHCK project put up posters in stores, carryouts, and recreation centers. Which of the following have you seen and/or read?” For each item, individuals were shown examples of materials used during the intervention and responded “yes,” “no,” or “maybe.” Because we developed a wide variety of handouts and posters throughout the intervention, we randomly chose a subset of examples in the IEQ to reduce respondent burden.

Only youths were asked about recreation center activities, such as cooking classes held in the center and frequency of recreation center attendance during the year of the intervention. Only adults were asked about participation in the BHCK text messaging and engagement with the social media programs during the year of the intervention, because these intervention components were targeted specifically at caregivers. Only when assessing exposure to the store component did we ask youths and adults to report the number of times they had shopped in the BHCK intervention corner stores and carryouts in the 7 days prior to the interview to improve the precision of reporting. We showed them pictures of the stores’ facades to aid with recall.

In addition, six red herring questions were used to address response bias. These included materials used in previous studies conducted in different sites. Respondents who answered positively to three (half) or more of the red-herring questions were excluded from the analysis.

#### Calculation of exposure scores

We calculated exposure scores for each intervention material or activity part of the BHCK program (seeing BHCK logo, seeing shelf label, participating in a taste test, seeing posters, seeing handouts, receiving giveaways, seeing educational displays, seeing a BHCK carryout menu, shopping in a BHCK store, attending a youth-led nutrition education session, interacting with BHCK youth-leaders, following or enrolling in BHCK social media or text messaging, and seeing BHCK social media posts). The detailed coding of the exposure scores is presented in Table 1. In short, as an example, for seeing materials or participating in taste tests, respondents had the option of answering “yes,” “no,” or “maybe” when shown a list of the materials followed by a picture of each item. For each question receiving a “yes” response one point was added, “maybe” was given 0.5 points, and “no” zero points.

**Table 1:** Exposure scores by BHCK intervention materials and activities

Intervention component	Intervention material or activity	Coding of exposure score
Corner stores and carryouts Recreation center Social media	Seeing BHCK logo in different places (stores, recreation centers, carryouts, and social media)	None = 0 1–2 places = 1.5 3–5 places = 4 6 or more = 6
Corner stores and carryouts	Seeing shelf label in different stores (BHCK corner stores and carryouts)	None = 0 1–2 places = 1.5 3–5 places = 4 6 or more = 6
Corner stores and carryouts Recreation center	Taste tests (10 questions) (and four cooking demos at recreation center – applied to child only)	For each taste test: Yes = 1 Maybe = 0.5 No = 0
Corner stores and carryouts Recreation center Social media	Posters (10 questions)	For each poster: Yes = 1 Maybe = 0.5 No = 0
Corner stores and carryouts Recreation center Social media	Handouts (nine questions)	For each handout: Yes = 1 Maybe = 0.5 No = 0
Corner stores and carryouts Recreation center	Giveaways (17 questions)	For each giveaway: Yes = 1 Maybe = 0.5 No = 0
Corner stores and carryouts Recreation center	Educational display (five questions)	For each display: Yes = 1 Maybe = 0.5 No = 0
Carryout only	Seeing redesigned menu (eight questions)	For each menu: Yes = 1 Maybe = 0.5 No = 0
Corner stores only	Purchased from a BHCK corner store in the past seven days	Continuous variable: total frequency of purchase summed for all stores (*n* = 21)
Recreation center (applied to child)	Average number of attendances in a BHCK recreation center (*n* = 7) during the intervention	Never = 0 <1 time/month = 0.5 1–3 time/month = 2 1–2 times/week = 6 >3 times/week = 12
Recreation center (applied to child)	Participation in a youth-led nutrition session	Continuous variable: total sessions attended (max: 14)
Corner stores and carryouts Recreation center (applied to child)	Interaction with BHCK youth leader	Never =0 1–5 times =1 6–10 times =2 >10 times = 3
Social media (applied to caregiver)	Followed or enrolled in BHCK social media (Facebook, Instagram, or texting)	For each account: Yes =1 No = 0
Social media	Seeing BHCK posts (Facebook or Instagram) (eight questions)	For each post: Yes = 1 No = 0
Overall BHCK exposure score		1. Summed points for each question for each intervention material and activity 2. Re-scaled exposure to materials and activities to range of 0–1 3. Summed all re-scaled exposure scores

We next added the points for each intervention component. For instance, we listed 10 possible examples of posters. These were added to give the total score for “seeing a poster” (possible range: 0–10 points). The highest total scores possible were 107 and 133 for caregivers and children, respectively. Because each intervention material or activity had a different number of questions and yielded different ranges in points, we re-scaled all scores (range 0–1) to give an equal weight to each component in the overall BHCK exposure score. For example, if a participant reported seeing 10 out of the 10 posters, their re-scaled exposure score was 1. If they reported seeing five posters, their re-scaled score was 0.5 points. Using methods like those previously published [13], the overall exposure score was calculated by summing the re-scaled exposure scores of the various BHCK intervention materials and activities. The highest re-scaled scores possible were 11 and 13 for caregivers and children, respectively, such that a 1-unit change in exposure represent a substantial difference in exposure to intervention activities.

#### Baseline individual and household sociodemographic characteristics

To address our third research question, we investigated whether the sociodemographic characteristics of the youths and caregivers could account for differences in exposure level that were not due to being assigned to treatment groups. We explored potential individual and household characteristics that could correlate with being exposed to the BHCK intervention, including child’s age and sex; caregiver’s age, sex, education level, and employment status; and household annual income, housing arrangement, and participation in a food assistance program. To collect this data, we used two instruments, the Child Impact Questionnaire [21, 22] and the Adult Impact Questionnaire [23]. Both questionnaires were adopted from similar instruments used in previous intervention trials in Baltimore City and are based on formative research [24, 25]. The Adult Impact Questionnaire included questions on demographics and household socioeconomic information, such as caregiver’s age, sex, education level, and employment status (currently employed, retired, disabled, or otherwise not employed); household annual income (categories US$0–10,000, 10,001–20,000, 20,001–30,000, or higher); housing arrangement; number of individuals living in the household (continuous variable); and participation in a food assistance program in the past 12 months [received WIC (Special Supplemental Nutrition Program for Women, Infants, and Children) or SNAP (Supplemental Nutrition Assistance Program) benefits].

### Data analysis

The statistical analysis of the data was conducted using software STATA 13.1 (College Station, TX, USA 2013). Means and standard deviations (SDs) were estimated for key baseline descriptors and exposure scores. Differences in continuous variables between the intervention and comparison groups were tested with an independent two-tailed *t*-test. A chi-square test for proportions was used to examine categorical variables.

Our model specification checks, including assessment of model residuals, revealed that treating our outcome (exposure score) as a continuous variable was problematic. Thus, we performed sensitivity analyses by fitting quadratic linear regression models (exposure scores were square-root transformed) to address the skewness of the dependent variable and to inform interpretation of the ordinal models (Additional file 2: Tables S1 and S2). We, therefore, used a categorical version, based on quartiles of the score, in all models.

Bivariate and multiple ordered logit regression models were used to analyze the association between youth and caregiver exposure levels (quartiles) and sociodemographic, household, and individual characteristics of the participants. Each model with exposure level (quartiles of exposure) was regressed on different independent variables:

- youth’s and caregiver’s ages (continuous variables)
- youth’s and caregiver’s sex
- caregiver’s educational level: categorized into less than high school, completed high school, and more than high school (for 2 than two years of college, associate degree, bachelor’s degree, or beyond) and coded as 0, 1, and 2, respectively
- housing arrangement: owned, rented, shared with family or other arrangement (group housing or transitional housing)
- number of individuals living in the household (continuous variable)
- participation in a supplemental nutritional assistance program: coded as 0 if not enrolled and 1 if enrolled in the past 12 months

The exposure level (outcome of interest) was stratified by quartiles (very low, low, medium, and high), in which we interpret the increase in each quartile as a higher level of exposure to BHCK activities. The final multiple ordinal regression model was selected based on the goodness of best fit using a stepwise backward method for the lowest Akaike information criterion.

The ordered logit model assumes that the effect of any of the independent variables is the same regardless of the level (quartile) of exposure (e.g., coefficients describing the relation between the lowest and all higher scores are the same as those describing associations between the next lowest and all higher scores) [26]. We calculated the variance inflation factor for each model to check for collinearity by performing a multiple linear regression, which were all below 1.0. The parallel assumption of the ordered logit regression was investigated by the likelihood-ratio test followed by the Brant test. Neither test rejected the null hypothesis that the coefficients were equal across quartiles of exposure. For all analyses, statistical significance was defined as *p* < 0.05.

## Results

### Characteristics of the baseline BHCK evaluation sample

No statistically significant differences were found between treatment groups at baseline (Table 2). The youths were on average 12 years old and most caregivers were female with an average age of 40. Most caregivers reported receiving SNAP in the past 12 months (70%). There were trends towards significance for a higher proportion of participants in the comparison group living with families compared to the intervention group (*p* = 0.1).

**Table 2:** Sociodemographic characteristics of the B’more Healthy Communities for Kids baseline evaluation sample

Baseline individual and household characteristics	*n*	Intervention group	Comparison group	*p* value
Youth		(*n* = 199)	(*n* = 186)	
Sex: female (%)	385	54.7	60.2	0.3
Age (years): mean (SD)	385	11.7 (1.4)	11.8 (1.6)	0.3
Caregiver and household		(*n* = 198)	(*n* = 188)	
Sex: female (%)	386	94.4	90.5	0.2
Age (years): mean (SD)	386	39.4 (9.1)	40.5 (9.7)	0.2
Education level				
<High school (%)	64	33	30	0.9
High school (%)	150	77	73	
>High school (%)	172	88	85	
Individuals in the household: mean (SD)		4.5 (1.5)	4.5 (1.6)	0.8
Annual income (US$)				
0–10,000 (%)	94	25.2	23.4	0.4
10,001–20,000 (%)	90	20.3	26.6	
20,001–30,000 (%)	60	17.2	13.8	
>30,000 (%)	142	37.4	36.2	
Food assistance participation				
SNAP (%)	274	71.2	70.7	0.9
WIC (%)	90	23.2	23.4	0.9
Housing arrangement				
Living with family^a^ or other^b^ (%)	39	7.1	13.1	0.1
Rented (%)	252	66.2	64.4	
Owned (%)	95	26.7	22.3	

*SD* standard deviation, *SNAP* Supplemental Nutrition Assistance Program, *WIC* Special Supplemental Nutrition Program for Women, Infants, and Children

^a^Living with family who own or rent the house

^b^Other included transitional housing or group house

### Patterns of dose received (exposure) by different components of the BHCK intervention

Some materials and activities of the BHCK trial appeared to have a higher mean exposure score than others. Caregivers were highly exposed to the BHCK logo, handouts, giveaways, and the social media program (Table 3).

**Table 3:** Caregiver exposure to the B’more Healthy Communities for Kids intervention materials and activities by intervention group (*n* = 386)

Caregiver exposure to BHCK materials and activities	Range	Intervention group	Comparison group	*p* value
		Mean ± SD	Mean ± SD	
Seeing BHCK logo in different places	0–1	0.31 ± 0.25	0.13 ± 0.20	<0.001
Seeing shelf label in different stores	0–1	0.07 ± 0.20	0.06 ± 0.21	0.7
Posters	0–1	0.13 ± 0.20	0.07 ± 0.14	0.001
Handouts^a^	0–1	0.20 ± 0.27	0.05 ± 0.13	<0.001
Giveaways^a^	0–1	0.22 ± 0.22	0.03 ± 0.10	<0.001
Educational displays	0–1	0.09 ± 0.18	0.07 ± 0.17	0.3
Seen redesigned menus^a^	0–1	0.15 ± 0.16	0.04 ± 0.12	<0.001
Taste tests	0–1	0.04 ± 0.12	0.05 ± 0.16	0.6
Purchased in different BHCK corner stores	0–1	0.07 ± 0.18	0.01 ± 0.08	<0.001
Followed or enrolled in social media^a^	0–1	0.21 ± 0.24	0.06 ± 0.14	<0.001
Seeing social media post	0–1	0.05 ± 0.13	0.03 ± 0.13	0.1
Overall BHCK exposure level^a^	0–12	1.60 ± 1.16	0.61 ± 1.0	<0.001

*BCHK* B’more Healthy Communities for Kids, *SD* standard deviation

^a^Statistically significant improvement in mean score from wave 1 to wave 2 (*p* < 0.05)

Although youths also appeared to be highly exposed to the BHCK logo and giveaways, exposure to educational displays was higher than other materials and greater than the mean exposure score among caregivers (Table 4).

**Table 4:** Youth exposure to the B’more Healthy Communities for Kids intervention materials and activities by intervention group (*n* = 385)

Youth exposure to BHCK materials and activities	Range	Intervention group	Comparison group	*p* value
		Mean ± SD	Mean ± SD	
Seeing BHCK logo in different places^a^	0–1	0.24 ± 0.3	0.13 ± 0.20	<0.001
Seeing shelf label in different stores	0–1	0.07 ± 0.20	0.03 ± 0.10	0.005
Posters^a^	0–1	0.15 ± 0.20	0.05 ± 0.10	<0.001
Handouts^a^	0–1	0.16 ± 0.23	0.04 ± 0.12	<0.001
Giveaways^a^	0–1	0.23 ± 0.24	0.06 ± 0.14	<0.001
Educational displays	0–1	0.17 ± 0.27	0.06 ± 0.19	<0.001
Seen redesigned menu	0–1	0.08 ± 0.20	0.04 ± 0.13	0.02
Taste test or cooking	0–1	0.12 ± 0.20	0.03 ± 0.02	<0.001
Youth-led nutrition education	0–1	0.05 ± 0.12	0.01 ± 0.09	0.03
Interaction with BHCK youth leader	0–1	0.08 ± 0.20	0.02 ± 0.10	0.001
Attended BHCK recreation center^a^	0–1	0.14 ± 0.22	0.01 ± 0.10	<0.001
Purchased in different BHCK corner stores	0–1	0.08 ± 0.20	0.02 ± 0.09	0.001
Seeing social media post	0–1	0.05 ± 0.18	0.02 ± 0.10	0.05
Overall BHCK exposure level^a^	0–13	1.6 ± 1.54	0.5 ± 0.83	<0.001

*BCHK* B’more Healthy Communities for Kids, *SD* standard deviation

^a^Statistically significant improvement in mean score from wave 1 to wave 2 (*p* < 0.05)

### Patterns of overall dose received (exposure) by BHCK intervention groups

**Fig. 2:**
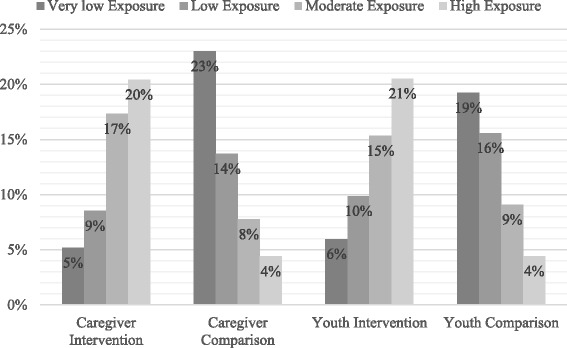
Youths’ and caregivers’ quartile of exposure level by intervention group. Total exposure score was stratified by quartiles (very low, low, medium, and high). Caregivers’ exposure levels of very low ranged from 0 to 0.25 with mean score of 0.08, low ranged from 0.27 to 0.75 with mean score of 0.48, medium ranged from 0.75 to 1.65 with mean score of 1.14, and high ranged from 1.67 to 6.76 with mean score of 2.79. Youths’ exposure levels of very low ranged from 0 to 0.18 with mean score of 0.04, low ranged from 0.20 to 0.62 with mean score of 0.36, medium ranged from 0.63 to 1.58 with mean score of 1.0, and high ranged from 1.60 to 7.57 with mean score of 3.0

The overall mean exposure to the BHCK intervention was low among adults (Table 3) and youths (Table 4). Despite the observed overall low level of exposure to the BHCK (youth: mean 1.1 points, SD ± 1.35, median 0.6, minimum 0 and maximum 7.6; caregiver: mean 1.1 points, SD ± 1.17, median 0.75, minimum 0 and maximum 6.7), in the intervention group, both youths and their caregivers demonstrated a significantly higher exposure level score than the control group. Figure 2 illustrates that although most participants in the intervention group had moderate to high exposure to BHCK, about 12% of the comparison group was moderate-to-highly exposed to the intervention.

### Patterns of dose received (exposure) by the two implementation waves

Preliminary information on the exposure level from wave 1 was used to inform the midcourse evaluation and to improve the reach and intensity of the overall program in wave 2. The midcourse evaluation allowed our research group to devote time and resources to make changes to the materials and intervention activities for wave 2. For instance, posters hung at community sites increased in size and were professionally redesigned to improve the visibility of the materials and intervention messages. Nutrition interaction sessions at corner stores, carryout restaurants, and recreation centers increased in intensity and duration. The quality of pictures and posting frequency were improved on social media platforms, and posts were boosted to the target audience (e.g., specific zip codes in Baltimore City). Finally, we mailed program flyers and promoted BHCK activities to participating households in the intervention during wave 2 to increase the reach and intensity of the promotional materials.

The second wave of the BHCK implementation had an overall higher exposure level score among the evaluation sample compared to the first wave: caregiver_wave 1_ 0.9 ± 1.1 vs caregiver_wave 2_ 1.3 ± 1.2, *p* < 0.001 (Table 3) and youth_wave 1_ 2.3 ± 3.4 vs youth_wave 2_ 3.8 ± 3.9, *p* = 0.003 (Table 4). Caregivers in wave 2 reported being more exposed to BHCK handouts, giveaways, social media posts, and the redesigned carryout restaurants menus than caregivers in wave 1 (Table 3). In wave 2, youths presented higher mean exposure score to the BHCK logo, posters, handouts, and giveaways, and reported attending more often an after-school program assigned to the BHCK intervention than youths in wave 1 (Table 4).

### Correlates of exposure to the BHCK intervention

The results of the bivariate ordered logit regression analysis between caregivers’ characteristics and quartiles of exposure level are presented in Table 5. We did not find any predictor that was correlated with caregivers’ exposure level in the unadjusted model. The adjusted analysis suggested that female caregivers had higher odds of exposure to the intervention (odds ratio OR 1.99; 95% confidence interval CI 1.05; 3.78) compared to male caregivers, after controlling for SNAP participation and housing arrangement.

**Table 5:** Caregivers’ correlates of level of exposure to the B’more Healthy Communities for Kids trial

Determinants of exposure to BHCK caregiver and household	Bivariate analysis		Final multivariable model^a^	
	Odds ratio (robust SE)	95% CI	Odds ratio (robust SE)	95% CI
Sex (reference: male)	1.81 (0.57)	(0.97; 3.36)	1.99 (0.65)	(1.05; 3.78)*
Age (years)	0.98 (0.01)	(0.97; 1.01)		
Education level				
<High school	Reference			
High school	0.95 (0.25)	(0.55; 1.62)		
>High school	1.01 (0.27)	(0.60; 1.70)		
Individuals in the household	0.98 (0.06)	(0.87; 1.10)		
Household annual income (US$)				
0–10,000	Reference			
10,001–20,000	0.87 (0.32)	(0.51; 1.51)		
20,001–30,000	0.88 (0.28)	(0.63; 1.87)		
>30,000	0.98 (0.06)	(0.80; 2.12)		
Food assistance participation				
SNAP (reference: non-SNAP)	0.71 (0.14)	(0.48; 1.07)	0.76 (0.15)	(0.51; 1.14)
WIC (reference: non-WIC)	0.90 (0.19)	(0.59; 1.37)		
Housing arrangement				
Living with family^b^ or other^c^	Reference			
Rented	0.93 (0.34)	(0.44;1.93)	0.95 (0.36)	(0.45; 1.99)
Owned	1.52 (0.62)	(0.69; 3.39)	1.51 (0.62)	(0.68; 3.37)

This is an ordered logistic regression on overall BHCK exposure level (quartiles) among adults

*BCHK* B’more Healthy Communities for Kids, *CI* confidence interval, *SE* robust standard error, *SNAP* Supplemental Nutrition Assistance Program, *WIC* Special Supplemental Nutrition Program for Women, Infants, and Children

*p < 0.5

^a^Final model selected based on goodness of best fit using a stepwise backward regression for the lowest Akaike information criterion: 1185.2

^b^Living with family who own or rent the house

^c^Other included transitional housing or group house

The bivariate analysis showed that youths’ age was significantly associated with odds of exposure. Specifically, the odds of exposure to the intervention decreased by 33% for each additional year of age (OR 0.77; 95% CI: 0.68; 0.86), and this association remained significant after controlling for household annual income and youths’ sex in the final multiple model (OR 0.77; 95% CI: 0.69; 0.88) (Table 6). In addition, youths in households with a higher annual income (>$30,000) appeared to be more likely to be exposed to the intervention than youths in the lowest income strata (OR 1.82; 95% CI: 1.13; 2.94), although this association did not remain significant after controlling for youth age and sex.

**Table 6:** Youths’ correlates of level of exposure to the B’more Healthy Communities for Kids trial

Determinants of exposure to BHCK youth	Bivariate analysis		Final multiple model^a^	
	Odds ratio (robust SE)	95% CI	Odds ratio (robust SE)	95% CI
Sex (reference: male)	0.74 (0.13)	(0.52; 1.06)	0.80 (0.14)	(0.56; 1.15)
Age (years)	0.77 (0.04)	(0.68; 0.86)*	0.77 (0.05)	(0.69; 0.88)*
Caregiver education level				
<High school	Reference			
High school	1.30 (0.32)	0.80; 2.11)		
>High school	1.37 (0.34)	(0.83; 2.25)		
Individuals in the household	0.95 (0.05)	(0.85; 1.05)		
Household annual income (US$)				
0–10,000	Reference		Reference	
10,001–20,000	1.29 (0.31)	(0.79; 2.09)	1.14 (0.29)	(0.69; 1.89)
20,001–30,000	1.66 (0.50)	(0.92; 3.01)	1.38 (0.43)	(0.74; 2.55)
>30,000	1.82 (0.44)	(1.13; 2.94)*	1.52 (0.37)	(0.94; 2.47)
Food assistance participation				
SNAP (reference: non-SNAP)	0.77 (0.16)	(0.51; 1.16)		
WIC (reference: non-WIC)	1.28 (0.29)	(0.81; 2.01)		
Housing arrangement				
Living with family^b^ or other^c^	Reference			
Rented	1.49 (0.43)	(0.85; 2.63)		
Owned	1.85 (0.63)	(0.94; 3.62)		

This is an ordered logistic regression on overall BHCK exposure level (quartiles) among youths

*BCHK* B’more Healthy Communities for Kids, *CI* confidence interval, *SE* robust standard error, *SNAP* Supplemental Nutrition Assistance Program, *WIC* Special Supplemental Nutrition Program for Women, Infants, and Children

^a^Final model selected based on goodness of best fit using stepwise backward regression for lowest Akaike information criterion: 1057.2

^b^Living with family who own or rent the house

^c^Other included: transitional housing or group house

## Discussion

To our knowledge, this is the first study to identify the patterns and determinants of the different levels of exposure to a MLMC childhood obesity prevention trial in a low-income urban setting. We described a detailed protocol of exposure scores evaluated through the IEQ instrument administered at post-intervention assessment for the intervention and control groups.

Exposure scores represented various levels of engagement with the intervention materials and activities, including seeing intervention materials, participating in educational sessions in stores and recreation centers, and receiving text messaging. Exposure to each intervention activity was then scaled (from 0 = not exposed through 1 = fully exposed), and summed to create an overall exposure score (dose received). Although combined exposure scores were low in both evaluation groups, the mean intervention exposure score was significantly higher among the intervention group than the comparison group for youths and caregivers. Low exposure scores have also been shown in previous community intervention trials, including the Navajo Healthy Stores [13] and in the BHEZ study conducted in urban Baltimore [15]. Our exposure analysis also indicated that the comparison sample was exposed to the intervention materials and activities, similar to what was reported in the Navajo Health Stores intervention [13].

We calculated the exposure score at the completion of wave 1 to inform process evaluation analysis, and this was key in comprehending which components of the intervention were successful and allowed the researchers to improve the intensity and reach of the communication materials and activities. These midcourse improvements between waves were reflected in our results, since handouts, posters, social media posts, and overall exposure had a slightly higher mean score in wave 2 compared to wave 1. Similarly in the literature, information collected on the exposure to the Active by Choice Today (ACT) and Pathways interventions are other examples of how a process evaluation may be used during the implementation of a program to improve the quality and acceptability of the trial among the target population [14, 27]. ACT used exposure findings for midcourse corrections, such as changes to the program curriculum, visual improvements to the program materials, and expansion of staff training to improve the intensity and dose of intervention over the course of the program [27]. Pathways used exposure results from the pilot testing phase to improve the family component of the intervention, as many children reported not attending the Family Fun Night in the intervention group and more than 40% of children in the control group reported exposure to half of the intervention items [28]. Our study supports previous literature indicating that a process evaluation may be used to improve the quality and dose of the intervention implementation and to ensure that it reaches the intended audience. Evaluating exposure during pilot or feasibility studies may provide critical information to researchers to avoid program contamination in the control group, and to maximize the reach and dose of large multi-level community trials [29].

A potential use of these exposure data is for impact analyses, as it may be hypothesized that individuals more exposed to a behavioral and environmental intervention will be more likely to have positive outcomes compared to those who were less exposed to the intervention over time [30]. In randomized controlled trials, this is also known as the treatment-on-the-treated effect (TTE), in which study participants are analyzed according to the treatment received, instead of the original treatment assigned (average treatment effects). Although this practice may violate randomization and increase potential biases, and the results may not infer the causal effect of the intervention, it is often used as a secondary evaluation analysis [31] and may provide an upper bound of program effectiveness [32]. For example, the BHEZ trial found that overweight girls who were more exposed to the intervention showed a 3.1 decrease in their BMI percentile over time compared to those with low exposure. A similar result in magnitude and strength was found in the average treatment effects analysis by treatment groups [15]. Another example is the CATCH study, which used the extent of the exposure to the intervention as a covariate to explain the change in the study outcome (serum cholesterol levels) and by substituting the indicator of study treatment with exposure levels as a TTE secondary analysis [32]. Due to the environmental nature of community-based interventions and the high likelihood of participant contamination, a TTE analysis using dose received information may generate an important estimate of the dose–response treatment effects.

Furthermore, it is important to recognize that participants’ sociodemographic characteristics may confound participation in the program activities (exposure), as well as be directly related to the final intervention outcome. We found that the sex of the caregiver, youth’s age, and household income were important factors that were associated with exposure to BHCK. Future analyses should link process evaluation information and outcome data to understand whether individual and household characteristics confound or mediate the relationship between exposure to the intervention and outcome changes in TTE analyses. In addition, a further TTE analysis using the score for exposure to the intervention should be interpreted cautiously as associations rather than intervention effects, while recognizing that confounding and selection bias may affect causal inference [33]. Understanding how a public health program reaches its target audience in the intended dose is critical, as community-based interventions are usually addressed to a large number of people. Thus, evaluating the interaction between the study participants and the program implementation informs the evaluation of the intervention, the representativeness of the study [34], and generates hypotheses for future research [11].

MLMC intervention trials face an additional challenge of partitioning out which specific intervention components had the greatest influence on individual outcomes [35]. However, incorporating exposure measures on outcome analyses may be another use of dose received in complex intervention trials analyses—one that has been used previously [30]. For example, would individuals who were highly exposed to posters and flyers be more likely to increase their knowledge of healthy eating than those with a lower exposure level to communication materials? Addressing this question would provide empirical evidence to the research community on the combination of intervention strategies that would best encourage behavior change among the targeted audience in the context of a multi-level intervention [36]. Lastly, community intervention trials often assume that the intervention protocol was implemented according to the initial standard, but programs are often adapted to the reality of the setting or not reach their intended target population, which may explain away treatment effects on an outcome of interest [30]. Therefore, assessing exposure and other process evaluation measures is essential to providing context to an intervention impact analysis.

A limitation of this study might be the risk of social desirability bias by treatment assignment, and by sex, age, and income, due to the self-report intervention exposure questionnaire. Some participants may have felt the need to inaccurately report that they have been exposed to certain activities or received BHCK materials. To address this issue, our questionnaire included red-herring questions to improve the validity of responses. Moreover, data collectors did not participate in the intervention implementation and were masked to the treatment group to avoid measurement error. Another limitation is that questions related to store purchasing behavior reflected the previous 7 days to the interview, which may have not reflected a habitual week of the interviewee, while other questions reflected the past year. Although the 7-day recall was conducted to improve quality of the report, as it relies on a participant’s memory, it may help explain the low exposure level to the intervention in this population. Future research may consider asking exposure questions consistently with the duration of the intervention program to best capture dose received. Furthermore, we did not assess the frequency of exposure to intervention materials (e.g., the amount of time a respondent saw a specific poster during the intervention or the different places the interviewee received a flyer) to minimize recall bias. However, future studies could consider assessing exposure in a longitudinal manner in a random sample (e.g., mid-intervention or after each intervention phase) to aid in midcourse correction and to inform intervention implementation. In addition, there was some overlap in the intervention and control zones, which might help to explain exposure to the intervention in the control group. However, despite this overlap, differences in exposure levels between the intervention and control group were still observed. Furthermore, including only a subset of the intervention communication materials in the questionnaire may have reduced the chance of some individuals recognizing the specific activities conducted during the intervention. Nevertheless, research staff chose examples of materials that represented the main topics covered during the intervention. Lastly, we classified exposure domains based on the multiple components of the BHCK program, and we assigned similar weights to each domain of interaction with the trial. However, behavior change is complex and occurs differently in subjects, as people may react in varying ways to activities and materials. Thus, differential exposure domains and weights could be assigned depending on the population, intervention, and context in which the program is being implemented.

## Conclusions

In conclusion, the BHCK intervention group was differentially exposed to the program components, and the comparison group also received some exposure, though to a lesser degree. The first wave experienced an overall lower score for exposure to the intervention by youths and caregivers compared to wave 2, highlighting the application of process evaluation findings to improve subsequent program reach and intensity. Future community-based environmental intervention trials may consider enrolling larger sample sizes and improving program intensity, as the likelihood of low exposure is high. Furthermore, samples should be selected to maximize exposure differences between the intervention and comparison groups and to minimize the potential for contamination to intervention activities. Our findings are important to implementation science, as they may inform pilot or feasibility trials of future large environmental community interventions prior to the implementation of the main program to better understand how the population perceives the activities and the dose and intensity needed in the setting.

Furthermore, our exposure analysis was key in identifying that some individual (age and sex) and household (income) characteristics correlate to exposure levels to the intervention. Future analyses linking exposure scores to the outcome should control for potential confounders in the TTE approach as a secondary evaluation assessment. Finally, achieving adequate exposure to the intervention from the participants’ perception of dose received is critical in environmental interventions to better understand the effectiveness of the intervention, as well as who was most likely to receive the treatment. This manuscript adds to implementation science by proposing a detailed protocol for the development of exposure scores (dose received) evaluated through process evaluation and by informing further impact analysis and intervention successes.

## Additional files

Additional file 1: CONSORT checklist of the B’more Health Communities for Kids program. (DOCX 158 kb)

Additional file 2: **Table S1.** Sensitivity analysis of caregiver correlates of level of exposure to the B’more Healthy Communities for Kids trial using a quadratic model for linear regression. **Table S2.** Sensitivity analysis of youth correlates of level of exposure to the B’more Healthy Communities for Kids trial using a quadratic model for linear regression. (DOCX 19 kb)

## Abbreviations

ACT: Active by Choice Today trial
BHCK: B’more Healthy Communities for Kids trial
BHEZ: Baltimore Healthy Eating Zones trial
BMI: Body mass index
CATCH: The Child and Adolescent Trial for Cardiovascular Health
CI: Confidence interval
IEQ: Intervention Exposure Questionnaire
MLMC: Multi-level multi-component
NICHD: National Institute of Child Health and Human Development
OR: Odds ratio
SD: Standard deviation
SE: Robust standard error
SNAP: Supplemental Nutrition Assistance Program
TTE: Treatment-on-the-treated effect
WIC: Special Supplemental Nutrition Program for Women, Infants, and Children
