# Supplementary material for: Exposure to a multi-level multi-component childhood obesity prevention community-randomized controlled trial: patterns, determinants, and implications
Source: Trials. 2018 May 22;19:287. doi: 10.1186/s13063-018-2663-y (PMC5964684; doi:10.1186/s13063-018-2663-y)
Supplement: Supplementary file 2 — Table S1. Sensitivity analysis of caregiver correlates of level of exposure to the B’more Healthy Communities for Kids trial using a quadratic model for linear regression. Table S2. Sensitivity analysis of youth correlates of level of exposure to the B’more Healthy Communities for Kids trial using a quadratic model for linear regression. (DOCX 19 kb) [file 13063_2018_2663_MOESM2_ESM.docx]

**Additional file 2**

**Table S1.** Sensitivity Analysis of Caregiver’s correlates of level of exposure to the B’more Healthy Communities for Kids trial using Quadratic Model for Linear Regression^1^

| Determinants of Exposure to BHCK Caregiver and Household | Bivariate Analysis | | | | Final Multiple Model^2^ | |
| --- | --- | --- | --- | --- | --- | --- |
|  | Coefficient (robust SE) | | 95% CI | | Coefficient (robust SE) | 95% CI |
| Sex (Reference: Male) | 0.17 (0.11) | | (-0.05; 0.39) | | 0.18 (0.11) | (-0.03; 0.41) |
| Age (years) | -0.01 (0.01) | | | (-0.01; 0.01) |  |  |
| Education Level |  | | |  |  |  |
| < High School | Reference | | |  |  |  |
| High School | 0.02 (0.08) | | | (-0.15; 0.20) |  |  |
| > High School | -0.03 (0.08) | | | (-0.19; 0.13) |  |  |
| Individuals in the household | | 0.01 (0.02) | | (-0.03; 0.04) |  |  |
| Household Annual Income (US$) |  | | |  |  |  |
| 0-10,000 | Reference | | |  |  |  |
| 10,001-20,000 | -0.12 (0.08) | | | (-0.28; 0.04) |  |  |
| 20,001-30,000 | -0.07 (0.09) | | | (-0.25; 0.11) |  |  |
| >30,000 | 0.01 (0.07) | | | (-0.14; 0.15) |  |  |
| Food Assistance Participation |  | | |  |  |  |
| SNAP (Reference: non-SNAP) | -0.06 (0.07) | | | (-0.18; 0.07) | -0.03 (0.06) | (-0.16; 0.09) |
| WIC (Reference: non-WIC) | -0.01 (0.07) | | | (-0.16; 0.12) |  |  |
| Housing Arrangement |  | | |  |  |  |
| Living w/ family^2^ or Other^3^ | Reference | | |  | Reference |  |
| Rented | -0.02 (0.09) | | | (-0.21; 0.16) | -0.04 (0.09) | (-0.24; 0.16) |
| Owned | 0.09 (0.11) | | (-0.11; 0.31) | | 0.07 (0.11) | (-0.14; 0.30) |

*Notes*: SE: robust standard error; CI: confidence interval; SNAP (Supplemental Nutrition Assistance Program); WIC (Special Supplemental Nutrition Program for Women, Infants, and Children); ^1^ Ordered Logistic Regression on overall BHCK exposure level (quartiles) among youth; ^2^ Final model selected based on goodness of best fit using stepwise backward regression for lowest Akaike information criterion (AIC): 1185.1; ^3^ Living with family who own or rent the house; ^4^ Other included: transitional housing or group house.

**Table S2.**  Sensitivity Analysis of Youth’s correlates of level of exposure to the B’more Healthy Communities for Kids trial using Quadratic Model for Linear Regression ^1^

| Determinants of Exposure to BHCK Youth | | Bivariate Analysis | | Final Multiple Model^2^ | |  |
| --- | --- | --- | --- | --- | --- | --- |
|  |  | Coefficient (robust SE) | 95% CI | Coefficient (robust SE) | 95% CI |  |
| Sex (Reference: Male) | | -0.09 (0.06) | (-0.22; 0.03) | -0.08 (0.06) | (-0.21; 0.04) |  |
| Age (years) | | -0.09 (0.02) | (-0.13; -0.05)* | -0.09 (0.02) | (-0.13; -0.04)* |  |
| Caregiver Education Level | |  |  |  |  |  |
| < High School | | Reference |  |  |  |  |
| High School | | 0.06 (0.09) | (-0.12; 0.25) |  |  |  |
| > High School | | 0.09 (0.09) | (-0.09; 0.27) |  |  |  |
| Individuals in the household | -0.02 (0.02) | | (-0.06; 0.02) |  |  | |
| Household Annual Income (US$) | |  |  |  |  |  |
| 0-10,000 | | Reference |  |  |  |  |
| 10,001-20,000 | | 0.07 (0.08) | (-0.11; 0.24) | 0.02 (0.09) | (-0.15; 0.20) |  |
| 20,001-30,000 | | 0.16 (0.09) | (-0.03; 0.36) | 0.10 (0.09) | (-0.09; 0.30) |  |
| >30,000 | | 0.17 (0.08) | (0.01; 0.33)* | 0.11 (0.08) | (-0.05; 0.26) |  |
| Food Assistance Participation | |  |  |  |  |  |
| SNAP (Reference: non-SNAP) | | -0.06 (0.07) | (-0.20; 0.07) |  |  |  |
| WIC (Reference: non-WIC) | | 0.05 (0.08) | (-0.09; 0.20) |  |  |  |
| Housing Arrangement | |  |  |  |  |  |
| Living w/ family^2^ or Other^3^ | | Reference |  |  |  |  |
| Rented | | 0.12 (0.10) | (-0.08; 0.32) |  |  |  |
| Owned | | 0.17 (0.11) | (-0.05; 0.40) |  |  |  |

*Notes*: SE: robust standard error; CI: confidence interval; SNAP (Supplemental Nutrition Assistance Program); WIC (Special Supplemental Nutrition Program for Women, Infants, and Children); ^1^ Ordered Logistic Regression on overall BHCK exposure level (quartiles) among youth; ^2^ Final model selected based on goodness of best fit using stepwise backward regression for lowest Akaike information criterion (AIC): 707.3; ^3^ Living with family who own or rent the house; ^4^ Other included: transitional housing or group house.
